# Regional Heterogeneity and Temporal Trends of Infant-Juvenile Paracoccidioidomycosis in Argentina: A 15-Year Comparative Study

**DOI:** 10.3390/jof12080568

**Published:** 2026-08-01

**Authors:** Gustavo Giusiano, Yone Chacón, Alejandra Aguilera, Diana Aguirre, Clarisa Aguirre, Martin Nazr Usandivaras, Teresa Corallo, Ruth Valdez, Lorena Damiana Altamirano, Andrea Vázquez, María de los Ángeles Sosa, Javier Mussin, Florencia Rojas

**Affiliations:** 1 Departamento Micología, Instituto de Medicina Regional, Universidad Nacional de Nordeste, CONICET, Resistencia 3500, Argentina; 2 Hospital Señor del Milagro, Salta 4400, Argentina; 3 Hospital Público Materno Infantil, Salta 4400, Argentina; 4 Hospital Pediátrico Avelino Castelán, Resistencia 3500, Argentina; 5 Hospital Pediátrico Juan Pablo II, Corrientes 3400, Argentina; 6 Hospital Materno Infantil Héctor Quintana, Jujuy 4600, Argentina; 7 Servicio de Microbiología, Hospital 4 de Junio Ramón Carrillo, Roque Sáenz Peña 3700, Argentina; 8 Laboratorio Central de Redes y Programas, Facultad de Ciencias Exactas y Naturales y Agrimensura, Instituto de Medicina Regional, Universidad Nacional del Nordeste, Corrientes 3400, Argentina

**Keywords:** *Paracoccidioides*, acute/subacute form, pediatric, clinical phenotypes, Argentina

## Abstract

Paracoccidioidomycosis (PCM) is a neglected systemic mycosis in Latin America. This study aims to characterize the epidemiology and clinical profiles of infant PCM cases (0 to 14 years old) over a 15-year period (2008–2023) in the two primary endemic regions of Argentina, the northeast (NEA) and the northwest (NWA) areas. Results reveal striking regional heterogeneity. Distribution showed clear disparities, 71.1% of cases were recorded in NWA, while 28.9% in the NEA area. The NWA region exhibited a constant temporal occurrence, whereas the NEA showed episodic occurrence/outbreaks potentially triggered by anthropogenic and climatic changes. Severe nutritional impairment and generalized lymphadenopathy were common clinical signs in both regions. Hepatosplenic enlargement and abdominal compromise with gastrointestinal manifestations dominated in the NWA (53.1%), while a higher prevalence of cutaneous/mucocutaneous (76.9%), pulmonary (38.4%) and osteoarticular (38.4%) manifestations characterized the clinical profiles in the NEA. These findings support the existence of distinct regional epidemiological and clinical phenotypes of pediatric PCM in Argentina. Recognizing these disparities is essential to improve clinical suspicion, diagnostic strategies, and the implementation of effective public health strategies for this severe pediatric disease.

## 1. Introduction

Although not formally declared, paracoccidioidomycosis (PCM) is a neglected disease and remains one of the most significant systemic mycoses in Latin America [1,2]. While the chronic adult clinical form is the most prevalent presentation, the acute/subacute form poses a significant challenge due to its rapid progression, severe general involvement and impairment, and high morbidity and mortality rates among children and young people [3,4].

Recent epidemiological shifts have been observed across the southern PCM endemic zone. Historically, two endemic areas have been delineated in Argentina. The more extensive zone, with a higher number of reported cases, is located in the northeast of the country (NEA), and the subtropical area of Las Yungas in northwest Argentina (NWA) [5]. Whether these geographically distinct endemic areas are associated with different clinical phenotypes remains poorly understood.

Emerging evidence indicates significant disparities between the NEA and the NWA regions regarding incidence rates, temporal patterns, and clinical manifestations particularly in pediatric cases [6]. Furthermore, variations in the epidemiology of acute/subacute infant-juvenile forms, were reported to be partly driven by climatic and anthropogenic changes as well as social and public health factors [7].

Understanding these regional heterogeneities is crucial to improving clinical suspicion, ensuring early diagnosis, optimizing therapeutic strategies, and implementing effective public health interventions for this highly vulnerable population.

Children and adolescents represent the population in which the acute/subacute form of PCM is characterized by rapid progression and substantially higher morbidity and mortality than the chronic adult form. Therefore, understanding the epidemiological and clinical characteristics of pediatric PCM is essential in order to improve early recognition and patient outcomes.

This study aims to characterize the epidemiology and clinical profile of infant PCM in the NEA and NWA regions of Argentina, seeking to provide a robust framework for the management of this neglected disease in the pediatric population.

## 2. Materials and Methods

A retrospective, comparative study was conducted over a 15-year period (2008–2023) to characterize the epidemiology of infant PCM in Argentina.

Data were retrieved from the Multicenter National Registry of PCM (MNR-PCM) which has been conducted since 2012, as well as from additional retrospective records. The MNR-PCM is coordinated by the Mycology Department, Instituto de Medicina Regional (IMR), Universidad Nacional del Nordeste (UNNE) in Chaco, Argentina. It includes referral hospitals located in endemic regions and major tertiary centers.

To identify regional variations, cases were stratified into the two Argentine endemic zones according to their different eco-epidemiological profiles. The extensive NEA region includes the provinces of Corrientes and Misiones, as well as portions of Chaco, Formosa, Santiago del Estero, Santa Fe, and Entre Ríos. The NEA endemic area has a warm subtropical climate without a dry season with a relief that varies from flat to undulating towards the east. Average annual temperatures range from 19 °C to 23 °C, with very long, humid, and hot summers, and average maximum temperatures ranging from 30 °C to 35 °C. Rainfall is distributed throughout most of the year, ranging from 1500 to 2000 mm annually. The NWA endemic area comprises only the Yungas mountain rainforest ecosystem (high-altitude jungle) in the provinces of Salta and Jujuy. The Yungas occupies mountain slopes between 700 and 1500 m and represents the altitudinal band with the highest rainfall (more than 2000 mm annually). The area has a humid subtropical climate with average annual temperatures ranging from 17 °C to 22 °C, with summer highs exceeding 30 °C [5,7,8].

### 2.1. Case Definition and Inclusion Criteria

The study included native patients (0 to 14 years old) with a confirmed diagnosis of PCM. Based on the EORTC (European Organization for Research and Treatment of Cancer) and the MSGERC (Mycoses Study Group Education and Research Consortium) consensus, the diagnostic confirmation was established by histopathology and/or mycological analysis of clinical specimens [9]. According to the global guideline for the diagnosis and management of the endemic mycoses and the Brazilian guidelines for the clinical management of PCM, a positive antibody detection using the Ouchterlony immunodiffusion (ID) assay was also included in the diagnostic criteria [10,11].

### 2.2. Data Collection and Variables

Epidemiological and clinical variables were extracted from medical records, documented on standardized forms and entered into a database to consolidate all information. The analyzed variables included: age at diagnosis, sex, province/locality of residence, migratory history (including the place of origin), tissue/organ involvement, clinical manifestation, and the diagnostic method used.

.

### 2.3. Severity Assessment

Clinical severity was stratified as moderate (without significant systemic dysfunction or life-threatening complications) or severe (profound systemic impairment) [12,13].

Severity categories were established based on clinical parameters traditionally associated with a poor prognosis. Severe forms were defined by the presence of 3 or more of the following findings: intense worsening of general and nutritional conditions (severe malnutrition/weight loss > 10%), generalized adenopathy (tumoral or suppurative), marked hepato- and/or splenomegaly, involvement of other organs (e.g., bone/skin) and high serum levels of specific antibodies.

When the worsening of general and nutritional conditions was absent or present (mild), the adenopathy was localized and nonsuppurative, absent or present (mild) hepato- and/or splenomegaly, with no involvement of other organs, and serum levels of specific antibodies were low, severity was classified as moderate.

### 2.4. Statistical and Temporal Analysis

Temporal data: The year of diagnosis was recorded to evaluate temporal trends and occurrence patterns.

Associations were evaluated using Fisher’s exact test. A significance level of 5% (*p* < 0.05) was considered statistically significant. The data analysis was performed using INFOSTAT (version 2017) statistical software [14].

A comparative approach was employed to identify regional disparities between the NEA and NWA. Geographical density analysis was conducted using a kernel density map to represent the spatial concentration of cases.

## 3. Results

### 3.1. Epidemiological Overview and Temporal Dynamics

**Figure 1:**
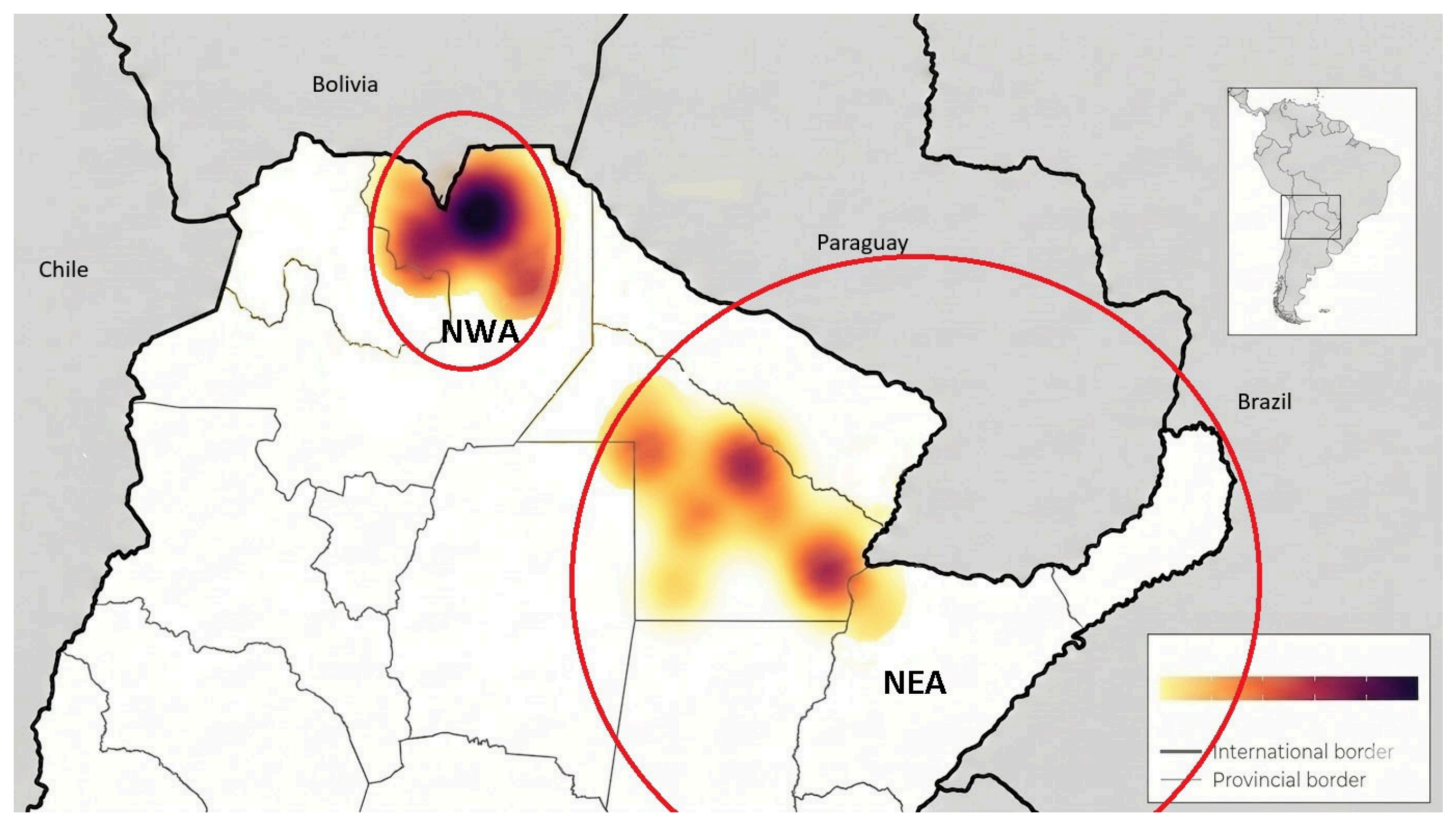
Heat map highlighting the spatial concentration of infant-juvenile Paracoccidioidomycosis cases in the northwest (NWA) and northeast (NEA) endemic areas of Argentina.

A total of 45 pediatric patients with PCM were analyzed between 2008 and 2023. The distribution revealed significant regional disparities, with 32 cases (71.1%) recorded in the NWA and 13 cases (28.9%) in the NEA area. All patients lived in rural communities within the endemic areas, distant from the major provincial capitals. As illustrated in Figure 1, geographical density analysis shows the spatial concentration of these cases within both eco-epidemiological zones.

**Figure 2:**
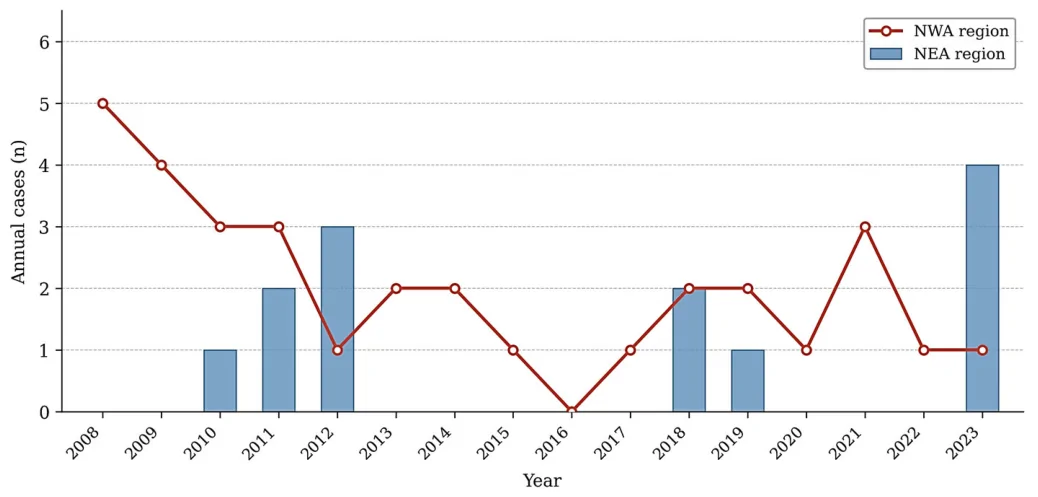
Time frame graph (2008–2023) showing the constant annual occurrence of infant-juvenile PCM in the NWA area versus the sporadic peaks of NEA region.

The temporal analysis identified divergent occurrence patterns in the two regions. The NWA region exhibited a constant temporal occurrence with cases reported nearly every year throughout the study period. In contrast, the NEA region showed episodic dynamic with periodic fluctuations or outbreaks. Notably, an initial cluster of six severe cases was detected in this region between 2010 and 2012, following years without any reported cases. See Figure 2.

The median patient age was 10 years (interquartile range IQR, 8–12 years), ranging from 1 to 14 years. A slight male predominance was observed (63.6% of cases), with a male-to-female ratio of 1.74:1.

### 3.2. Clinical Manifestations and Regional Phenotypes

**Figure 3:**
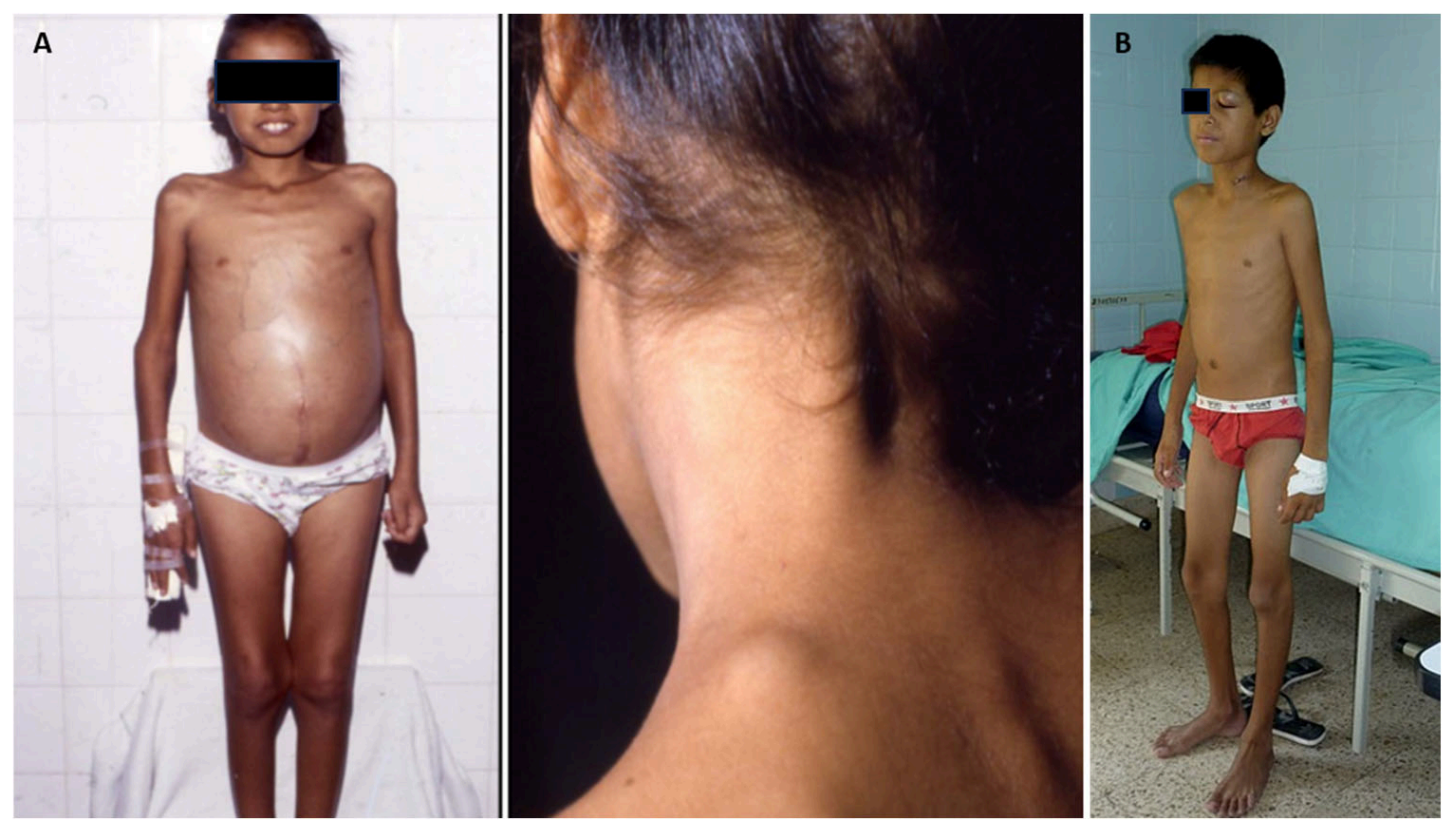
(**A**): Weight loss, abdominal compromise and cervical and supraclavicular lymph node involvement observed in a girl from NWA region. (**B**): A 10-year-old boy from the NEA region presenting with severe nutritional impairment and a subcutaneous lump on his left eyelid.

Fever, asthenia and anemia were recorded in all children. Weight loss and lymph node involvement were the predominant clinical findings in both endemic areas, affecting nearly all patients (Figure 3). Specifically, weight loss was documented in 100% of NWA cases and in 92.3% of NEA cases, while lymphadenopathy was observed in 96.9% and 92.3% of patients, respectively.

**Figure 4:**
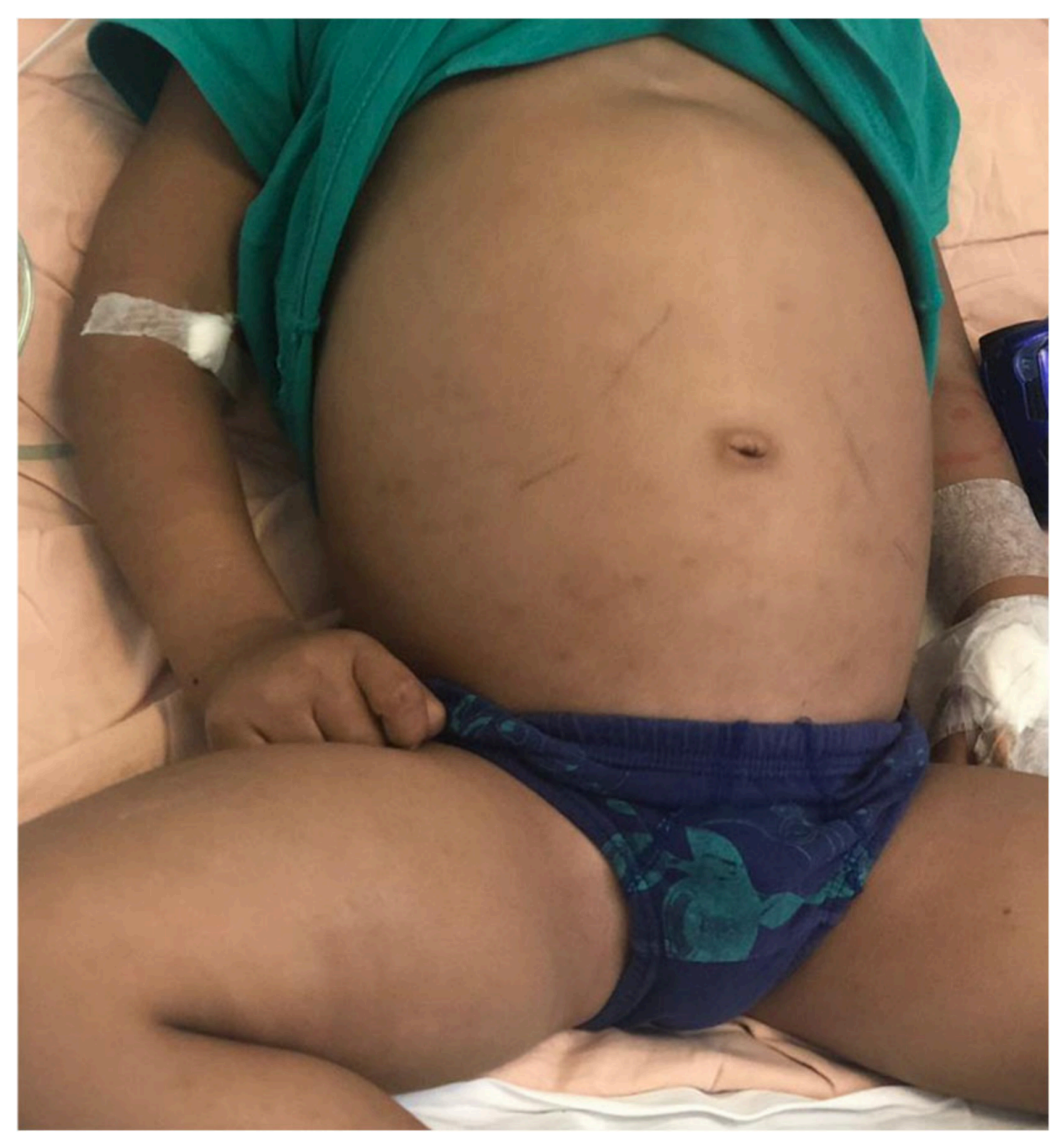
A distended abdomen with hepatosplenomegaly, typically observed in children from NWA endemic area.

In contrast, marked regional heterogeneity emerged regarding organ-specific manifestations. Patients from NWA predominantly exhibited hepatosplenic enlargement and abdominal compromise (ascites), along with gastrointestinal manifestations including diarrhea (53.1%, reported in 17/32 cases), constituting the hallmark clinical phenotype of this region (Figure 4).

**Figure 5:**
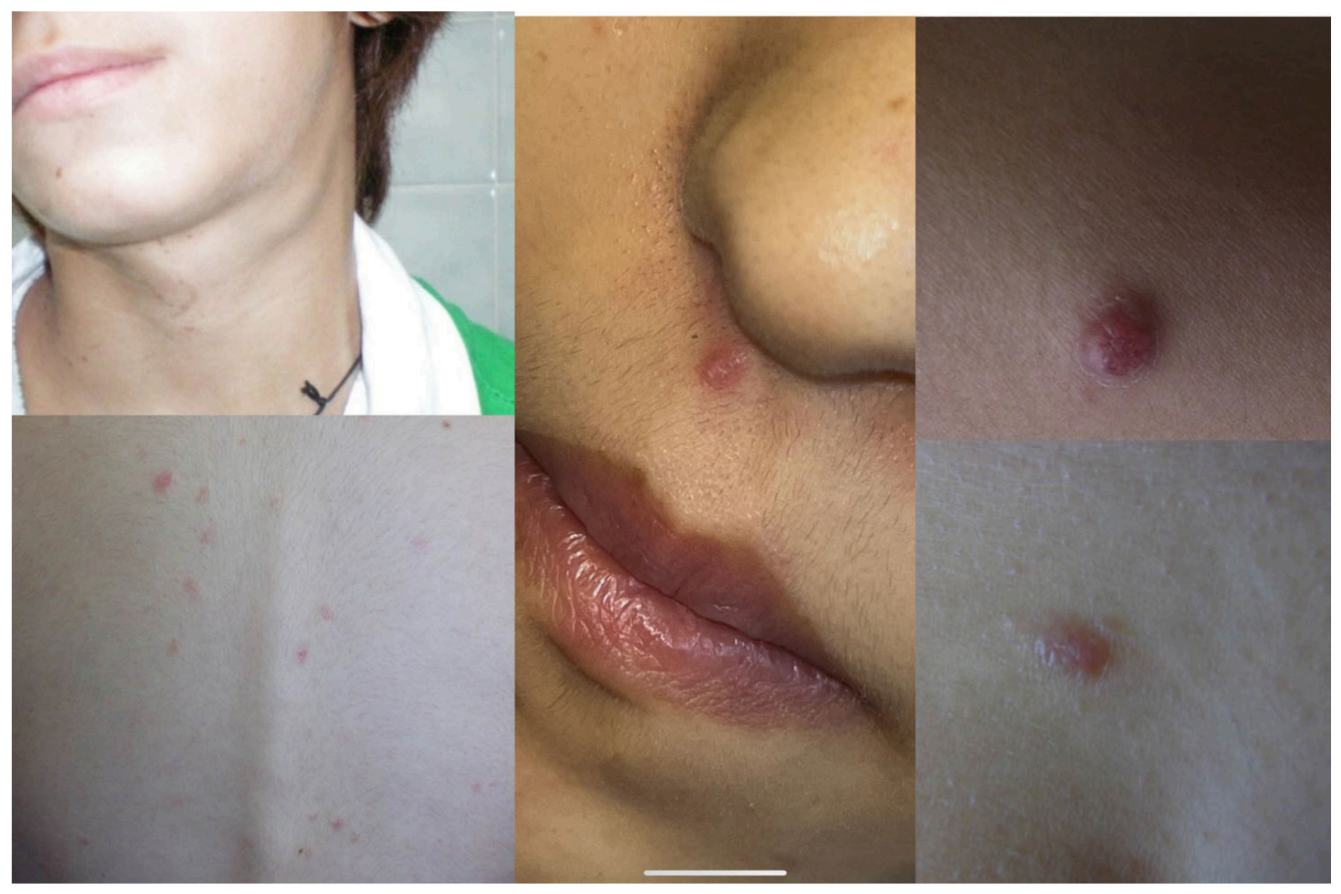
Localized adenopathy and cutaneous papules observed in patients from NEA region.

Cutaneous and mucocutaneous lesions were significantly more frequent in the NEA than in the NWA cohort (76.9% vs. 9.4%; *p* < 0.001). These manifestations included cutaneous abscesses, papules and oral mucosal lesions, representing one of the most distinctive regional differences identified in the study (Figure 5).

Pulmonary involvement was proportionally more frequent in NEA patients than in NWA cases (38.4% vs. 9.4%). Bone involvement also showed regional variation, occurring in 38.4% of NEA patients compared with 6.2% of NWA cohort. Although pulmonary and osteolytic involvement were more prevalent in NEA patients, these differences did not reach statistical significance, likely due to the limited sample size.

**Figure 6:**
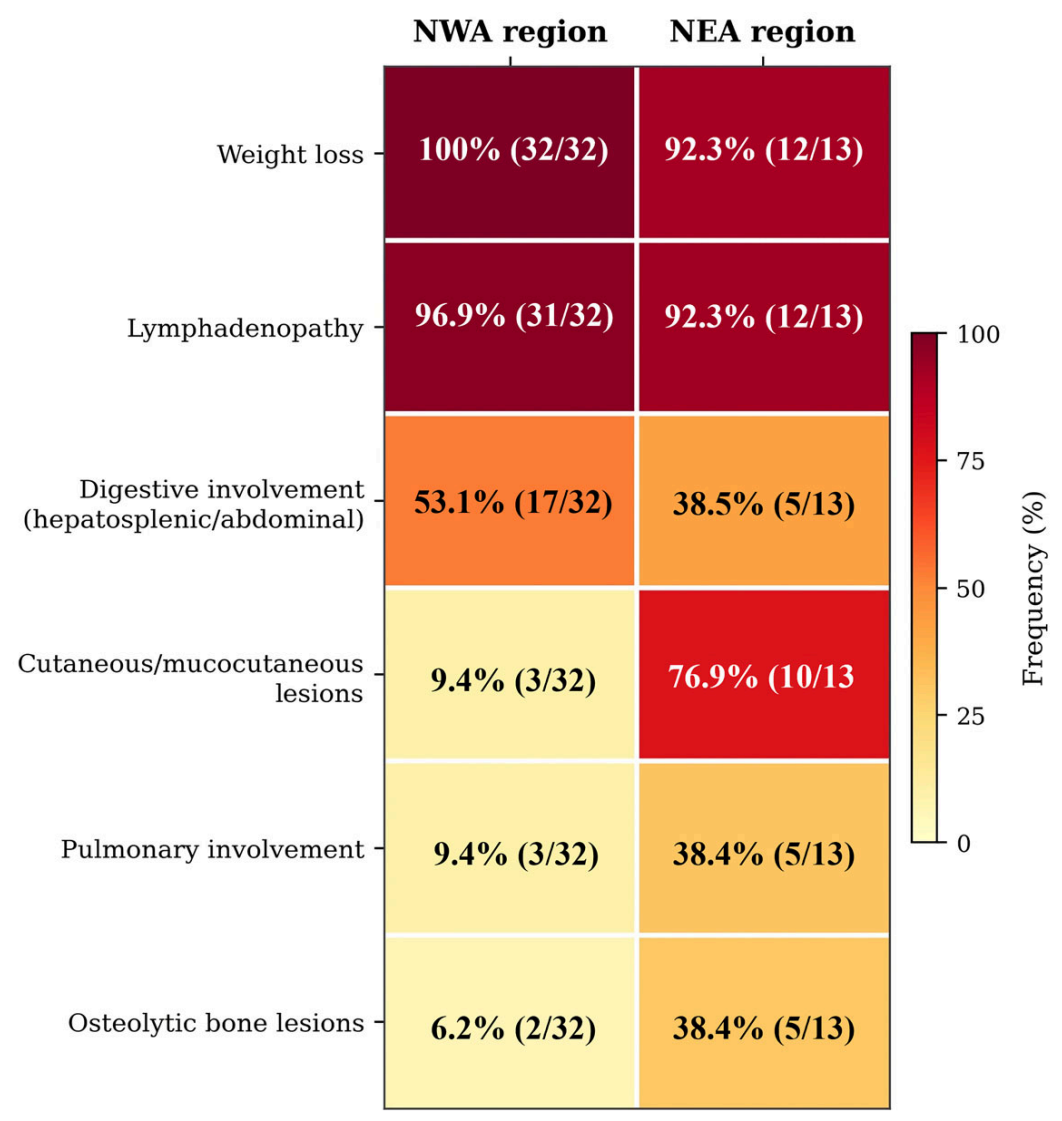
Main clinical manifestation distribution registered in pediatric population with paracoccidioidomycosis from the northwest (NWA; *n* = 32) and northeast (NEA; *n*= 13) endemic regions of Argentina. Bars represent the percentage of patients presenting each clinical manifestation. Comparisons between groups were performed using Fisher’s exact test. No post hoc analyses were required.

The main clinical manifestations and the heterogeneity observed are represented in Figure 6.

### 3.3. Severity Assessment and Nutritional Status

According to the predefined criteria, most pediatric patients (91.1%) presented with severe disease in both endemic regions (see Table 1). Severe disease was defined by significant weight loss (>10%), generalized adenopathy, and/or visceral involvement and/or high serum levels of specific antibodies ranging from 1/16 to 1/256. Weight loss was the predominant finding (Figure 3), and showed a strong correlation with the severe clinical forms. While weight reduction was observed in nearly all patients, emaciation was the true marker of severity.

While most cases were classified as severe, a marginal trend towards a higher proportion of moderate presentations was observed in the NEA group, although without reaching statistical significance (23.1% vs. 3.1% in NWA; *p* = 0.056). Clinical severity in both cohorts demonstrated a strong correlation with the degree of malnutrition, specifically emaciation, as a consistent marker of severe systemic compromise.

## 4. Discussion

### 4.1. Eco-Epidemiological and Socioeconomic Context of the Studied Areas

The highest frequency of pediatric cases targeted by this study were recorded primarily in the rural areas of the Chaco province in the NEA and the Salta province in the NWA area. These territories are characterized by profound structural inequalities. While official Argentine urban indicators (EPH-INDEC) restrict provincial metrics to the urban agglomerations of the main administrative capitals, peripheral and rural districts in the provinces of Chaco and Salta exhibit chronic socioeconomic vulnerability. According to regional multidisciplinary matrices, structural poverty rates in these areas sometimes exceeded 46% during the period 2008–2023. These areas are defined by subsistence farming, high levels of informal employment, and a critical deficit in basic infrastructure, particularly the lack of safe water networks [15,16,17].

### 4.2. Regional Heterogeneity and Epidemiological Patterns

The first national multicenter study on PCM, published in 2023, revealed clear disparities in clinical and demographic features between the two geographic regions, highlighting the occurrence of acute/subacute forms exceeding 37% in the NWA. Despite their clinical relevance and longstanding recognition, comparative studies between these two endemic poles of Argentina had not previously been conducted [6]. Our study confirms the persistence of two geographically distinct endemic regions and reveals significant regional heterogeneity between the two areas, which are only 500 km apart. It also suggests distinct ecological differences and, potentially, particular host–pathogen interactions.

In the NWA, PCM exhibits a constant temporal occurrence, suggesting the persistence of a stable ecological niche in which *Paracoccidioides* remains constantly present in the environment. Conversely, the NEA region shows episodic dynamics, characterized by periods without reported cases interspersed with temporal clusters since 2010; previously only one case had been reported. This pattern has previously been associated with ecological disturbances driving episodic outbreaks in the NEA area [7]. Climatic and anthropogenic changes disturb the natural habitat of the fungus increasing human exposure. In the NEA, intense deforestation and the ecological impact of hydroelectric dams have altered soil and air humidity. Additionally, enhanced human infections and clusters of acute/subacute juvenile forms have been reported in correlation with ENSO (The El Niño-Southern Oscillation)-related climatic anomalies occurring one to two years prior cases detection [7,18,19,20]. Unlike the NWA, where the Yungas mountain rainforest provides a relatively stable ecological environment, the NEA is one of the Argentine areas most strongly affected by ENSO, with marked increases in precipitation, flooding, and soil humidity during and after El Niño episodes. These environmental disturbances may favor fungal persistence, growth, or dispersion, and increase human exposure. The first cluster observed in the present study (2010–2012) corresponds to the outbreak previously reported following the 2009–2010 El Niño event [7].

Subsequent temporal fluctuations (in 2018–2019 and 2023) may have also been influenced by climatic variability, as a strong El Niño event was registered in 2015–2016 [21]. However, the descriptive nature of the present study and the limited number of pediatric cases preclude establishing a formal temporal association.

### 4.3. The Triad of Vulnerability: Poverty, Malnutrition, and Host Susceptibility

The marked predominance of severe clinical forms (91.1%) observed in our pediatric cohort underscores a close synergy between socioeconomic deprivation, nutritional status, and environmental exposure. In the endemic rural areas of the Salta and Chaco provinces, the likelihood of developing active and progressive PCM in this population is modulated by a dual mechanism: high environmental exposure to *Paracoccidioides* propagules and a concomitant failure of host cellular immunity driven by chronic malnutrition and systemic poverty. Regarding exposure, pediatric patients are integrated into rural microenvironments where anthropogenic landscape modifications, such as intense deforestation, facilitate aerosolization and inhalation of infectious conidia. However, environmental exposure alone does not fully explain the clinical aggressiveness and rapid visceral dissemination documented in these patients.

Severe protein–energy malnutrition (weight loss > 10%), observed in nearly all cases, acts as a critical predisposing factor that impairs cellular immunity which essential for containing *Paracoccidioides* spp. infection, and has been considered a critical predisposing condition to developing severe PCM. Aggressiveness and high mortality rates have been reported for this clinical form enhanced by severe malnutrition [3,13,22]. This synergy between systemic poverty and nutritional deficiency is probably the major driver that facilitates the progression from latent infection to severe, potentially explaining the aggressive phenotype and the prevalence of infant-juvenile PCM observed in this study. Emaciation was the first clinical sign described in all patients from NWA and 91.7% from the NEA region.

In these areas, this physiological vulnerability is exacerbated as a direct consequence of the structural poverty. Geographic isolation and the lack of infrastructure, such as access to safe drinking water, lead to recurrent diarrhea episodes, enteroparasitosis and secondary malabsorption syndromes, leaving the host immunologically defenseless.

The province of Salta is home to one of the most diverse indigenous populations in Argentina. The northern region, where the highest frequency of cases in this study was recorded, has the largest concentration of indigenous communities in Argentina. Other communities are located in Chaco province. Also noteworthy is the correlation between the impact of deforestation on traditional diets and the forced nutritional transition of these communities toward malnutrition [16].

The socioeconomic vulnerability of the target population in both areas partly determines the likelihood of falling ill. Limited economic resources negatively affect not only the host’s nutritional status and immune response to fungal infections but also hinder early detection due to limited access to a public or private health system capable of providing a timely diagnosis, which is generally far from the area where these patients live [2,23]. Due to the large vulnerable population, the lack of PCM prevention strategies and the limited availability of diagnostics and treatments in endemic zones, *Paracoccidioides* was included in the World Health Organization’s (WHO) list of fungal priority pathogens [24]. The severity of our cases, mostly classified as severe due to profound systemic impairment and malnutrition, reflects the vulnerability of the pediatric population, aggravated by socioeconomic factors since limited resources hinder early access to the public health system.

### 4.4. Clinical Phenotypes and Diagnostic Challenges

Although lymphatic involvement and weight loss were the most common clinical manifestations in both regions, significant regional differences in the frequency and distribution of organ-specific manifestations were observed. NWA patients predominantly presented with visceral involvement, including liver and spleen compromise and digestive symptoms such as diarrhea and ascites. In combination with pre-existing malnutrition, this creates a synergistic effect resulting in severe and profound systemic worsening. In contrast, NEA patients exhibited a broader spectrum of manifestations, particularly characterized by higher frequencies of cutaneous/mucocutaneous, pulmonary, and bone compromise. In this region, infant-juvenile PCM has emerged episodically with periodic fluctuations of low frequency or outbreaks since 2010. The lack of clinical awareness due to its recent emergence in the NEA, coupled with the magnitude of the lymphadenopathy, often delays diagnosis. Lymphomas or other malignancies are ruled out first before considering any fungal etiology. In NWA, the clinical profile often mimics visceral leishmaniasis, a co-endemic disease.

These findings demonstrate substantial regional heterogeneity in the clinical expression of infant-juvenile PCM in Argentina, supporting the concept that geographically distinct endemic areas may present different clinical phenotypes within the same disease entity. Further analysis and studies are required to determine the reason or reasons for these clinical and epidemiological differences between the two endemic regions.

### 4.5. Hypothesizing Genetic and Immunological Drivers

We already analyzed that, according to the severity assessment criteria, the severe forms were characterized by a profound systemic deterioration with intense malnutrition, and this is an important contributing factor. The aggressive nature of the disease observed in very young infants (from a 1-year-old in NWA) may suggest host-related vulnerability. Immunogenetic variability influences the immune response to *Paracoccidioides* infection and could partially explain interindividual differences in disease severity. However, since the present study did not evaluate genetic or immunological parameters, this hypothesis requires further multidisciplinary research.

Beyond individual susceptibility, another potential factor contributing to the distinct clinico-epidemiological patterns observed between the two endemic regions may involve differences in host immunogenetic background and population ancestry [25]. Although both the NEA and NWA include indigenous communities, the NWA has one of the highest Native American ancestral components reported in Argentina, reflecting the long-term continuity of Andean indigenous populations. In contrast, the NEA has a different demographic history and admixture profile, influenced by distinct indigenous groups and subsequent migration patterns [16,26,27]. Since host immunogenetic factors influence susceptibility and clinical expression of PCM [28], it is plausible that regional differences in host immunogenetic profiles could partially modulate susceptibility, inflammatory patterns, or organ tropism. This hypothesis is supported not only by the regional differences observed in the present study but also by those previously identified in the nationwide multicenter study conducted in Argentina [6]. Although the present study did not evaluate genetic markers, this hypothesis warrants investigation in future multidisciplinary studies integrating epidemiological, immunological, and genomic approaches.

The regional heterogeneity between NWA and NEA cases identified in this study may reflect the interaction of multiple environmental, ecological, sociocultural, and host-related factors. Several important questions regarding the epidemiology of PCM remain unresolved. Although important progress has been made in recent years, current knowledge on the geographic distribution of the cryptic *Paracoccidioides* species and their clinical relevance or potential association with different clinical presentations remains limited [29].

## 5. Conclusions

This study demonstrates that infant-juvenile PCM in Argentina is not a clinically or epidemiologically homogeneous entity, but rather a complex disease with marked regional heterogeneity. While the NWA maintains a constant and predictable temporal occurrence, the NEA has emerged as a region of episodic occurrences driven by significant anthropogenic and climatic shifts, strongly influenced by the ENSO phenomenon.

Although severe nutritional deterioration and generalized lymphadenopathy are common clinical hallmarks in both endemic areas, distinct regional phenotypes were identified. The NWA region exhibited a comparatively more homogeneous clinical profile dominated by constitutional symptoms, generalized lymphadenopathy and hepatosplenic and abdominal involvement. In contrast, the NEA region showed a broader and more polymorphic dissemination pattern characterized by higher frequency of cutaneous/mucocutaneous and pulmonary compromise and osteolytic bone lesions.

These findings suggest that the clinical phenotypes observed across both endemic regions may result from a multifactorial interaction involving environmental exposure, ecological transformation, socioeconomic vulnerability, healthcare accessibility, and potentially host-related immunogenetic determinants.

Recognition of these regional particularities is essential to improve early clinical suspicion, optimize diagnostic strategies, and develop region-specific public health interventions for one of the most severe and neglected pediatric systemic mycoses in Latin America.

Future multidisciplinary research is required to fully elucidate the mechanisms underlying the regional heterogeneity of pediatric PCM in Argentina.

## Figures and Tables

**Table 1:** Distribution of patients according to the severity assessment evaluation.

Severity Classification	NEA	NWA
Moderate	3 (23.1%)	1 (3.1%)
Severe	10 (76.9%)	31 (96.9%)

NEA: northeast Argentina; NWA: northwest Argentina.

## Data Availability

Please add the corresponding content of this part.
